# The Risk of Hippocampal Metastasis and the Associated High-Risk Factors in 411 Patients With Brain Metastases

**DOI:** 10.3389/fonc.2022.808443

**Published:** 2022-02-14

**Authors:** Peng Xie, Hui Qiao, Huiling Hu, Wenlong Xin, Huanyu Zhang, Ning Lan, Xiaohua Chen, Yan Ma

**Affiliations:** ^1^ Department of Radiation Oncology, The First Hospital of Lanzhou University, Lanzhou, China; ^2^ Department of Oncology, The First Hospital of Lanzhou University, Lanzhou, China; ^3^ Department of Radiology, The First People’s Hospital of Lanzhou City, Lanzhou, China; ^4^ Department of Radiology, The First Hospital of Lanzhou University, Lanzhou, China

**Keywords:** brain metastasis, hippocampal metastasis, radiation therapy, hippocampal avoidance, whole-brain radiation therapy (WBRT)

## Abstract

**Background and Aims:**

To retrospectively analyze the incidence of hippocampal metastasis and the associated high-risk factors in patients with brain metastases and evaluate the safety of hippocampal avoidance whole-brain radiation therapy (HA-WBRT).

**Methods:**

We retrospectively analyzed the data of patients with brain metastases diagnosed by contrast-enhanced cranial Magnetic resonance imaging (MRI) at the First Hospital of Lanzhou University from 2017 to 2020. The boundaries of the hippocampus, hippocampus + 5 mm area, hippocampus + 10 mm area, and hippocampus + 20 mm area were delineated, and the distances from the brain metastases to the hippocampus were measured. Univariate and multivariate logistic regressions were adopted to analyze the high-risk factors of hippocampal metastasis.

**Results:**

A total of 3,375 brain metastases in 411 patients were included in the analysis. The metastasis rates in the hippocampus and surrounding areas of the entire group were as follows: 7.3% (30/411) in the hippocampus, 16.5% (68/411) in the hippocampus + 5 mm area, 23.8% (98/411) in the hippocampus + 10 mm area, and 36.5% (150/411) in the hippocampus + 20 mm area. Univariate logistic regression showed that the pathological type, the number of metastases, the maximum diameter of metastases, and the volume of brain metastases were all correlated with hippocampal metastasis. Multivariate logistic regression showed that the pathological type, the number of metastases, and the total volume of metastases were correlated with hippocampal metastasis.

**Conclusion:**

The pathological type, the number of metastases, and the total volume of metastases are the high-risk factors associated with hippocampal metastasis. Small cell lung cancer (SCLC) has a significantly higher rate of hippocampal metastasis than other tumor types. The greater the number and total volume of metastases, the more likely the hippocampal metastasis. For patients with SCLC or a greater number and total volume of brain metastases, the implementation of HA-WBRT may bring a higher risk of tumor recurrence.

## Introduction

As a regular site of malignant tumor metastasis, the brain metastasis rate in tumor patients is over 20% (1). Lung cancer is the most common (30% to 50%) primary cancer for patients with brain metastases (1, 2). Other than that, breast cancer also often metastasizes to the brain (3). Radiation therapy plays a vital role in treating brain metastases, one widely used type of which is whole-brain radiation therapy (WBRT). With the advancement of radiation therapy and medical treatment, the survival time of patients with brain metastases has been greatly prolonged. Reportedly, the median survival time of patients with brain metastases from EGFR-positive lung cancer could reach 25 months (4). As more and more patients began to pursue a higher quality of life, the neurotoxicity of WBRT became a public concern. However, the probability of cognitive dysfunction after receiving WBRT can reach 50% to 90%, which significantly affects patients' quality of life (5). In the meantime, the hippocampus is a crucial component of the temporal lobe closely related to memory (6) and learning functions (7, 8). WBRT could reduce hippocampal neurogenesis and dendritic spine density, resulting in impaired learning and memory functions (9). Studies have confirmed that hippocampus avoidance WBRT (HA-WBRT) could reduce the risk of cognitive impairment (10, 11). Yet, the safety of HA-WBRT depends on the incidence of brain metastases in the hippocampus and its surrounding areas. Studies have reported hippocampal metastasis rates of different tumors between 1.7% and 4.6%, and that of the hippocampal + 5 mm area between 3.1% to 12.2% (12–16). Nevertheless, the results in the existing studies vary considerably, and the applicability of HA-WBRT in patients with primary tumors such as SCLC or a large number of brain metastases is still controversial. Hence, studying the probability of hippocampal metastasis in patients with brain metastases is necessary to better inform the choice of WBRT or HA-WBRT for the patients. In this study, the safety of HA-WBRT was explored by retrospectively analyzing the incidence and associated high-risk factors of hippocampal metastasis in a large sample of patients with multi-tumor brain metastases.

## Materials and Methods

### Patient Screening

This study included 411 patients with brain metastases admitted to our hospital from 2017 to 2020, following the inclusion and exclusion criteria below. Patients pathologically diagnosed as malignant tumors and diagnosed as brain metastasis by contrast-enhanced MRI were included. Patients a) with two or more malignant tumors, b) incomplete medical records, or c) other craniocerebral diseases were excluded.

### Brain Metastasis Location and Hippocampus Delineation Method

**Figure 1:**
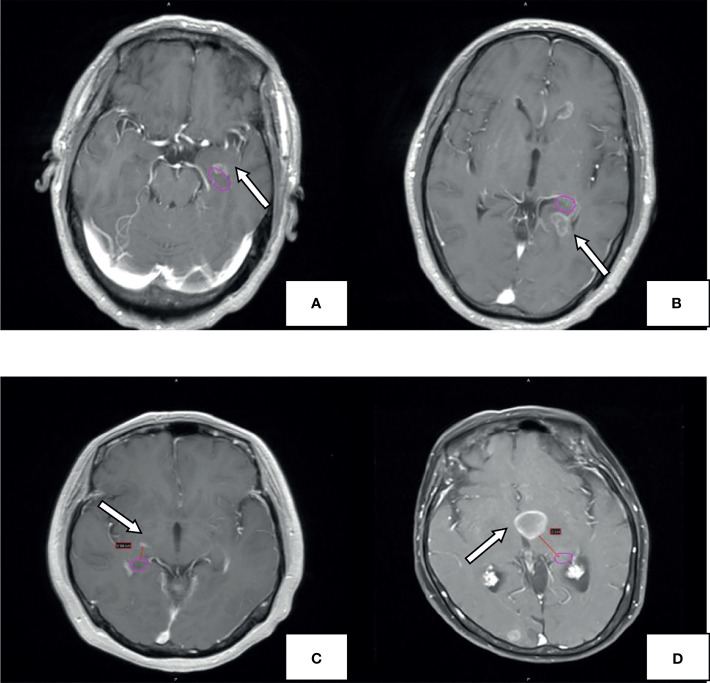
Example for hippocampal metastasis. The pink circle represents the hippocampal contour; the arrow points to the metastasis. Brain metastases invade the hippocampus **(A)**, hippocampus+5mm area **(B)**, hippocampus+10mm area **(C)**. hippocampus +20mm area **(D)**.

The patients' head MRI data were acquired through the hospital's PACS system. The number and distribution of brain metastases were observed from the T1-weighted contrast-enhanced images. The brain is divided into the frontal lobe, parietal lobe, temporal lobe, occipital lobe, cerebellum, insular lobe, and brainstem according to the anatomical boundaries. All brain metastases were delineated after importing the contrast-enhanced MRI data into the Varian Eclipse Treatment Planning System. The anatomical boundaries of the hippocampus were delineated according to the standard established in RTOG 0933. Then, the boundaries were pushed outwards in every direction to get the boundaries of the hippocampus + 5 mm area, hippocampus + 10 mm area, and hippocampus + 20 mm area, respectively. Finally, the shortest distances between the boundaries of brain metastases and that of the hippocampus were measured. The location of brain metastases and the hippocampus is shown in **Figure 1**.

### Research Content

The incidence of brain metastases in the hippocampus and surrounding areas was calculated. The differences in hippocampal metastasis rates among different tumor types were analyzed. Statistical methods were employed to determine the relationship between hippocampal metastasis and various clinical features so that the high-risk factors for hippocampal metastasis can be screened out.

### Statistical Methods

Logistic regression was conducted in SPSS 21 to analyze the correlation between hippocampal metastasis and the various clinical factors, including age, gender, pathological type, number of brain metastases, the maximum diameter of metastases, and total volume of metastases. The optimal cutoffs for the number of brain metastases, the maximum diameter of metastases, and the total volume of metastases were obtained by calculating the area under the receiver operating characteristic (AUROC) curve. The difference was considered statistically significant with P < 0.05.

The protocol of this retrospective study was approved by the ethics committee of the First Hospital of Lanzhou University (LDYYLL-2021-346).

## Results

The 411 eligible patients included in the analysis consist of 249 males and 162 females. The ages of the patients were between 18 and 86, and the median age was 62. The primary cancers of the patients included 341 (83.0%) lung cancers, 30 (7.3%) breast cancers, and 40 (9.7%) other cancers. As shown in **Table 1**, the whole group of patients had 3,375 brain metastases, averaging 8.2 per patient, and over 20% of the patients had 10 or more brain metastases. The most frequent location of brain metastasis was the frontal lobe, followed by the parietal lobe, cerebellum, temporal lobe, and occipital lobe. The metastasis rate to the brainstem and the insular lobe was very low (below 3%). The distribution of brain metastases in the intracranial substructures is shown in **Table 2**. The metastasis rates in the hippocampus and surrounding areas of the entire group are as follows: 7.3% (30/411) in the hippocampus, 16.5% (68/411) in the hippocampus + 5 mm area, 23.8% (98/411) in the hippocampus + 10 mm area, and 36.5% (150/411) in the hippocampus + 20 mm area. Tumors of different pathological types have varied metastasis rates to the hippocampus. The metastasis rates in the hippocampus and surrounding areas of patients with small-cell lung cancer (SCLC) were the highest, reaching 18.1% (19/105) in the hippocampus and 22.9% (24/105) in the hippocampus + 5 mm area. The metastasis of different tumors in the hippocampus and surrounding areas is shown in **Table 3**. Univariate logistic regression showed that the pathological type, the number of metastases, the maximum diameter of metastases, and the volume of brain metastases were correlated with hippocampal metastasis (**Table 4**). Multivariate logistic regression showed that the pathological type, the number of metastases, and the total volume of metastases were correlated with hippocampal metastasis (**Table 5**).

**Table 1:** Patients characteristics (n = 411).

Parameters	Numbers of patients	Percentage (%)
Sex		
Male	249	60.6
Female	162	39.4
Age in years		
<60	184	44.8
≥60	227	55.2
Median		62
Range		18–86
Primary tumours		
Lung cancer	341	83.0
Non-small cell lung cancer	236	57.4
Small cell lung cancer	105	25.5
Breast cancer	30	7.3
Others	40	9.7
Number of BMs		
1–3	212	51.6
4–9	105	25.5
≥10	94	22.9
Maximum diameter of BMs (mm)		
≤20	279	67.9
>20	132	32.1
Aggregate volume of BMs (mm³)		
≤7400	271	65.9
>7400	140	34.1

**Table 2:** The distribution of metastatic lesions by intracranial location (*n* = 3375).

Intracranial location	Number of lesions	Percentage (%)
Frontal lobe	798	23.6
Parietal lobe	712	21.1
Temporal lobe	626	18.5
Occipital lobe	477	14.1
Cerebellum	666	19.7
insular lobe	22	0.7
Brainstems	74	2.2
Total	3375	100

**Table 3:** Metastasis rates of hippocampi and surrounding areas in different pathological types of tumors.

Primary tumours	hippocampal (%)	hippocampal+5mm (%)	hippocampal+10mm (%)	hippocampal+20mm (%)
Lung cancer				
Non-small cell lung cancer	3.8 (9/236)	14.8 (35/236)	22.9 (54/236)	36.4 (86/236)
Small cell lung cancer	18.1 (19/105)	22.9 (24/105)	31.4 (33/105)	40 (42/105)
Breast cancer	3.3 (1/30)	10 (3/30)	13.3 (4/30)	30 (9/30)
Others	5 (2/40)	17.5 (7/40)	20 (8/40)	30 (12/40)

**Table 4:** Univariate analysis of the risk factors of hippocampal involvement with different margin definition.

Characteristics	HMs			HMs+5mm			HMs+10mm			HMs+20mm		
	OR	95%CI	P	OR	95%CI	P	OR	95%CI	P	OR	95%CI	P
Age (years)												
Continuous variable	0.986	0.953-1.021	0.435	1.002	0.978-1.027	0.876	1.002	0.981-1.024	0.832	1.000	0.981-1.019	0.979
<60	1			1			1			1		
≥60	0.901	0.428-1.896	0.783	1.146	0.676-1.946	0.641	1.196	0.752-1.903	0.450	1.144	0.757-1.727	0.523
Sex												
Male	1			1			1			1		
Female	0.980	0.458-2.094	0.958	1.151	0.676-1.958	0.605	1.033	0.646-1.652	0.892	1.263	0.832-1.917	0.272
Primary tumour												
1.NSCLC	1			1			1			1		
2.SCLC	5.309	2.292-12.296	0.000	1.691	0.935-3.058	0.082	1.508	0.891-2.551	0.126	1.174	0.723-1.906	0.517
3.BC	0.824	0.101-6.741	0.857	0.621	0.178-2.162	0.454	0.509	0.170-1.525	0.228	0.742	0.325-1.697	0.480
4.others	1.257	0.261-6.047	0.775	1.185	0.485-2.897	0.709	0.827	0.359-1.905	0.655	0.742	0.358-1.538	0.423
Number of BM												
Continuous variable	1.028	1.012-1.045	0.001	1.072	1.047-1.098	0.000	1.084	1.056-1.114	0.000	1.139	1.096-1.185	0.000
<3	1											
≥3	3.986	1.492-10.642	0.006									
<10				1			1					
≥10				6.353	3.613-11.170	0.000	6.225	3.709-10.449	0.000			
<4										1		
≥4										5.269	3.348-8.294	0.000
Maximum diameter of BM												
Continuous variable	2.007	1.281-3.142	0.002	1.046	1.025-1.067	0.000	1.047	1.027-1.067	0.000	1.040	1.022-1.058	0.000
≤20	1											
>20	5.188	2.347-11.466	0.000									
≤12				1								
>12				3.441	1.921-6.162	0.000						
≤16							1					
>16							2.886	1.796-4.639	0.000			
≤15										1		
>15										2.347	1.541-3.577	0.000
Volume of BM												
≤7400	1											
>7400	9.570	3.804-24.007	0.000									
≤4457				1			1			1		
>4457				7.748	4.116-14.588	0.000	4.939	2.995-8.146	0.000	3.956	2.563-6.106	0.000

**Table 5:** Multivariate analysis of the risk factors of hippocampal involvement with different margin definition.

Characteristics	HMs			HMs+5mm			HMs+10mm			HMs+20mm		
	OR	95%CI	P	OR	95%CI	P	OR	95%CI	P	OR	95%CI	P
Primary tumour												
1.NSCLC	1			1			1			1		
2.SCLC	6.016	2.428-14.905	0.000	2.140	1.067-4.293	0.032	1.835	1.002-3.359	0.049	1.478	0.836-2.611	0.179
3.BC	0.887	0.101-7.789	0.914	0.772	0.196-3.033	0.711	0.570	0.172-1.892	0.358	0.794	0.319-1.979	0.621
4.others	0.926	0.179-4.791	0.927	0.965	0.356-2.615	0943	0.653	0.256-1.663	0.372	0.629	0.275-1.435	0.270
Number of BM												
<3	1											
≥3	3.469	1.187-10.139	0.023									
<10				1			1					
≥10				4.625	2.475-8.640	0.000	4.974	2.801-8.833	0.000			
<4										1		
≥4										5.000	3.071-8.141	0.000
Maximum diameter of BM												
≤20	1											
>20	1.419	0.418-4.826	0.575									
≤12				1								
>12				0.782	0.307-1.993	0.607						
≤16							1					
>16							1.137	0.492-2.623	0.764			
≤15										1		
>15										0.783	0.347-1.769	0.557
Volume of BM												
≤7400	1											
>7400	6.265	1.611-24.364	0.008									
≤4457				1			1			1		
>4457				7.198	2.762-18.758	0.000	3.634	1.558-8.475	0.003	4.789	2.5132-10.757	0.000

## Discussion

According to the results of a study by Sun (2019) on 116 Chinese patients with brain metastases from multiple cancers, the metastasis rate in the hippocampus was 1.7%, and the metastasis rate in the hippocampus + 5 mm area was 11.2% (13). Based on data of 226 Chinese patients with brain metastases, Han reported that the metastasis rate in the hippocampus + 5 mm area was only 3.1%. However, Han's study did not include patients with SCLC (15). A study with a larger sample of 371 patients conducted in the United States by Gondi found that the metastasis rate in the hippocampus + 5 mm area was 8.6% (16). These findings suggest a low metastasis rate in the hippocampus and surrounding areas, i.e., HA-WBRT is safe. However, the results of this study showed that the metastasis rates in the hippocampus and hippocampus + 5 mm area were 7.3% and 16.5%, respectively, which was higher than those in previous studies. The reason may be the high proportion of patients with multiple brain metastases in this study. Univariate and multivariate logistic regressions in this study showed that the number of brain metastases was an independent risk factor for hippocampal metastasis, which was consistent with previous studies. For example, Chen suggested that the number of brain metastases was a high-risk factor for hippocampal metastasis, and patients with a large number of metastases were more susceptible to hippocampal metastases (12). Guo pointed out that the risk of hippocampal metastasis was significantly higher if the number of brain metastases was equal to or above 5 (17). The average number of brain metastases in the 411 patients in this study was 8.2 (411/3,375); patients with no less than 10 brain metastases accounted for 22.9% (94/411); patients with no less than 20 brain metastases accounted for about 10% (41/411). In other similar studies, the average number of brain metastases in the included patients was only 3 to 5, and patients with no less than 10 brain metastases accounted for about 10% (12–16). The higher hippocampal metastasis rate in this study may be due to the larger number of patients with multiple metastases included herein.

In addition, subgroup analysis of this study found that the metastasis rate in the hippocampus and surrounding areas of 105 patients with brain metastasis from SCLC were significantly higher than other tumor types such as non-small cell lung cancer (NSCLC) and breast cancer (BC). The hippocampal metastasis rate of SCLC patients (18.1%) was over twice that of the entire group of patients (7.3%). SCLC patients accounted for over 25% (105/411) of the entire group, which may be the reason for the high hippocampal metastasis rate in this study. Univariate and multivariate logistic regressions also found that the pathological type was correlated with the risk of hippocampal metastasis. The risk of SCLC metastasis in the hippocampus was 6 times that of NSCLC. Many previous studies also found that SCLC patients seemed to have a higher rate of hippocampal metastasis. Harth reported that the hippocampal metastasis rate of SCLC patients could reach 18.2%, and the metastasis rate in the hippocampal + 5 mm area was 27.2%, but only 11 SCLC patients were included in that study (18). Kirakli also reached a similar conclusion that the hippocampal metastasis rate of 54 SCLC patients was 32% (19). Chen reported that the hippocampal metastasis rate in 90 SCLC patients was 7.8%, and the metastasis rate in the hippocampal + 5 mm area was 14.4% (12). Compared with other tumors, SCLC has a higher rate of hippocampal metastasis, which may be attributed to its higher malignancy and brain metastasis tendency. Studies have shown that the brain metastasis rate in SCLC patients at first diagnosis was 10%, about 40% to 50% during diagnosis and treatment, and 60% to 80% in patients surviving for over 2 years (20). Brain metastases in SCLC patients occur significantly earlier than patients with other tumors, and more brain metastases can be clinically observed in the hippocampus. However, reports on the hippocampal metastasis rate of SCLC patients vary. Some reported that the hippocampal metastasis rate of SCLC patients was 5%, and the metastasis rate in the hippocampal + 5 mm area was 12.2% (17). Zhao showed that the metastasis rate in the hippocampus + 5 mm area of SCLC patients was 5.9%, which was relatively low (21). The results of these studies differ. The RTOG 0933 (10) suggested that adopting HA-WBRT could significantly reduce the incidence of cognitive dysfunction, which was confirmed in subsequent studies (11, 22). However, it has been reported recently that compared with traditional prophylactic cranial irradiation (PCI), hippocampus avoidance PCI (HA-PCI) did not show better protection for neurocognitive functions (23, 24). Considering these controversial results, it is safe to conclude that the hippocampal metastasis rate of SCLC patients is significantly higher than that of patients with other tumors, and HA-WBRT may bring a higher risk of tumor recurrence for these patients, thus should be used with caution. Although some guidelines recommend HA-WBRT for SCLC patients undergoing PCI (25, 26), this practice is clearly controversial.

In the research on factors of hippocampal metastasis, many have reported the number of brain metastases as an influencing factor (12, 15, 17). Sun came to a similar conclusion in the study of patients with brain metastases from BC, i.e., the risk of hippocampal metastasis increased significantly with the number of brain metastases (27). Univariate logistic regression in this study showed that the pathological type, the number of metastases, the maximum diameter of metastases, and the volume of brain metastases were all correlated with hippocampal metastasis. Further multivariate logistic regression showed that the pathological type, the number of metastases, and the total volume of metastases were independent high-risk factors of hippocampal metastasis, which was consistent with previous studies. This study also found that the risk of hippocampal metastasis increased 3 times if the number of brain metastases was no less than 3. However, when calculating this result, the optimal cutoff (2.5) for the number of brain metastases obtained by using the hippocampus as the diagnostic outcome corresponded to an AUROC value of only 0.679 (<0.7), suggesting that the reliability may be poor. With no less than 10 brain metastases, the risk of metastasis in hippocampus + 5 mm area and hippocampus + 10 mm area increased by 4 times; with no less than 4 brain metastases, the risk of metastasis in hippocampus + 20 mm area increased by 5 times; When calculating the optimal cutoffs for these subgroups, the corresponding AUROC values were all above 0.7, suggesting higher reliability. Studies have also pointed out that the volume of brain metastases is correlated with hippocampal metastasis (16). Analysis in this study also indicated a higher metastasis rate in the hippocampus and surrounding areas of patients with a higher total volume of brain metastases. For example, when the total volume of metastases is above 7400 mm³, the risk of hippocampal metastasis increases 6-fold; when the total volume of metastases is above 4457 mm³, the risk of metastasis in the hippocampus + 5 mm, hippocampus + 10 mm, and hippocampus + 20 mm increased 7-fold, 3-fold, and 4-fold, respectively. Therefore, it is not difficult to understand the correlation between the total volume of brain metastases and the risk of hippocampal metastasis. The main reason is that cases with larger total metastasis volume include two scenarios: the number of metastases is small, but the individual lesions are large; the metastases are small but numerous in the whole brain. A larger metastasis has a shorter distance to the hippocampus, thus being more likely to invade it. A greater number of metastases produce a greater chance for lesions to land in the hippocampus and surrounding areas. Therefore, the total metastasis volume is a good indicator of these two scenarios. In addition, previous studies have identified age as an independent risk factor affecting hippocampal metastasis (28), and patients over 60 had an increased risk of hippocampal metastasis (29). However, factors such as age and gender were not found to correlate with the hippocampal metastasis rate in this study.

As an analysis based on a large sample of patients with brain metastasis from multiple tumors, a higher proportion of patients with multiple brain metastases were included in this study, which may better reflect the risk of hippocampal metastasis in patients with brain metastases. This study has certain reference values for studying the characteristics of brain metastasis and the probability of hippocampal metastasis in the Chinese population. However, as a retrospective analysis, this study has certain limitations, and bias is inevitable in the selection and collection of cases. For example, this study included 40 patients with primary cancers located outside the lung and the breast. However, those lesions covered 8 histological types, i.e., kidney cancer, pancreatic cancer, colorectal cancer, esophageal cancer, gastric cancer, nasopharyngeal cancer, ovarian cancer, and endometrial cancer, and the brain metastasis rate in the hippocampus + 5 mm area of this subgroup was as high as 17.5%, which may have impacted the results.

## Conclusion

In conclusion, the risk of hippocampal metastasis is related to the pathological type, the number of brain metastases, and the total volume of metastases. Patients with SCLC have a significantly higher hippocampal metastasis rate than those with other tumors. When the number of brain metastases was no less than 10, the chance of metastasis in the hippocampus + 5 mm and hippocampus + 10 mm regions increased more than 4-fold; when the total volume of metastases was above 7400 mm³, the risk of hippocampal metastasis increased more than 6-fold. Therefore, for patients with SCLC or a greater number and total volume of brain metastases, the implementation of HA-WBRT may bring a higher risk of tumor recurrence, thus warrants caution.

## Data Availability Statement

The data analyzed in this study is subject to the following licenses/restrictions: Any request for data and material may be sent to the first author or corresponding author. Requests to access these datasets should be directed to PX, 1005443068@qq.com.

## Ethics Statement

The protocol of this retrospective study was approved by the local ethics committee of the First Hospital of Lanzhou University (LDYYLL-2021-346).

## Author Contributions 

PX: Data curation, Manuscript writing. HQ: Research designing, Manuscript revising. HH, WX, HZ, NL, XC, and YM: Date collection. All authors contributed to the article and approved the submitted version.

## Conflict of Interest

The authors declare that the research was conducted in the absence of any commercial or financial relationships that could be construed as a potential conflict of interest.

## Publisher’s Note

All claims expressed in this article are solely those of the authors and do not necessarily represent those of their affiliated organizations, or those of the publisher, the editors and the reviewers. Any product that may be evaluated in this article, or claim that may be made by its manufacturer, is not guaranteed or endorsed by the publisher.

## Abbreviations

HA-WBRT, hippocampal avoidance whole-brain radiation therapy; SCLC, Small cell lung cancer; WBRT, whole-brain radiation therapy; NSCLC, non-small cell lung cancer; BC, breast cancer; PCI, prophylactic cranial irradiation; HA-PCI, hippocampus avoidance PCI.
